# *Pternopetalum paucifoliolatum* (Apiaceae), a new Critically Endangered species from Sichuan, China

**DOI:** 10.3897/phytokeys.166.54646

**Published:** 2020-11-12

**Authors:** Jian-Fei Ye, Liang Chen, Zhang-Jian Shan, Xiao-Jie Li, Ce-Hong Li

**Affiliations:** 1 Beijing Botanical Garden, Institute of Botany, Chinese Academy of Sciences, Beijing 100093, China; 2 State Key Laboratory of Systematic and Evolutionary Botany, Institute of Botany, Chinese Academy of Sciences, Beijing 100093, China; 3 College of Life Sciences, University of Chinese Academy of Sciences, Beijing 100049, China; 4 Guangxi Guijiang Forestry Surveying & Planning & Designing Co., Ltd, Nanning 530022, China; 5 Emei Mountain Biotic Resource Experimental Station, Emeishan 614201, China

**Keywords:** Conservation, limestone, rare species, Umbelliferae

## Abstract

*Pternopetalum paucifoliolatum*, a new species from Sixigou Scenic Area, Emeishan City, Sichuan Province, is proposed and described. Diagnostic morphological characters, full description, detailed illustrations, and a distribution map are provided. The new species is similar to *P. porphyronotum* in possessing the 1-pinnate leaves and the abaxially purple-red leaflets, but differs from the latter by shorter stature, fewer leaflets ((1–) 3–7) and rays (5–8), the leaflet margin white-ciliate. The new species, which is assessed as Critically Endangered (CR), was only found on limestone cliffs. We also provide a new key to the species of *Pternopetalum*.

## Introduction

*Pternopetalum* Franch. (Apiaceae), including ca. 20 species, is endemic to east Asia and one of the largest genera of Apiaceae in Asia (Pimenov et al. 2004; Wang 2012). It is distributed in South Korea, Japan, China, and the adjacent eastern Himalayan regions (Pu 1985; Pimenov and Leonov 1993; Pu and Phillippe 2005), with a diversity center in the East Himalaya-Hengduan Mountains region (Su and Sheh 2001). This genus is characterized by petals saccate at base, umbellules with only 2–4 (–5) flowers, and styles and rays reflexed in fructescence (Pu 1985; Pu and Phillippe 2005; Wang 2012). After Wang’s (2012) revision of *Pternopetalum*, four new species of this genus were described (Bhaumik and Satyanarayana 2014; Tan et al. 2014, 2015; Zhong et al. 2018).

During a field investigation in Sixigou Scenic Area, Emeishan City, Sichuan Province, China, in March 2019, we found an unusual *Pternopetalum* population with flowers. We noticed that they not only have dwarf plants, lobe margin white-ciliate, but also have 1-pinnate leaves with few leaflets, differing from all other known species of the genus. We revisited the same locality and collected several specimens with fruit in May 2020. Thus, this dwarf species with (1–) 3–7 leaflets is described here as new to science.

### Key to the species of *Pternopetalum*

**Table:** 

1	Stout perennial, usually more than 30 cm high; stylopodium conic; styles erect, twice as long as the stylopodium; calyx evident, triangular or subulate	**2**
–	Slim perennial or annual, usually not more than 30 cm high; stylopodium lower conic; styles reflexed at upper part, shorter or equal than stylopodium at length; calyx minute or obsolete	**10**
2	Stem well developed; umbels terminal and lateral	**1. *P. delavayi***
–	Stem dwarf; umbels terminal, occasionally with one or two lateral umbels	**3**
3	Leaves only basal	**4**
–	Leaves basal and cauline, occasionally cauline leaves absent	**6**
4	Leaves ternate; leaflets 3, ovate, margins crenate	**2. *P. nudicaule***
–	Leaves ternate, 2–4-pinnate or finely dissected	**5**
5	Leaves finely dissected; ultimate segments linear	**3. *P. trichomanifolium***
–	Leaves bipinnate; ultimate segments ovate	**4. *P. bipinnatum***
6	Leaves 2-ternate or ternate-2-pinnate; ultimate segments acute at apex	**7**
–	Leaves 2-ternate or ternate-1-pinnate; ultimate segments caudate at apex	**5. *P. rosthornii***
7	Leaves ternate; leaflets 3, ultimate leaflets usually 2–3(–5)-lobed	**6. *P. vulgare***
–	Leaves 2-ternate or ternate-2-pinnate; ultimate segments margins incised-serrate or without lobed	**8**
8	Leaves ternate-2-pinnate; ultimate segments margins incised-serrate	**7. *P. latipinnulatum***
–	Leaves 2-ternate; ultimate segments without lobed	**9**
9	Leaf blades subleathery; ultimate segments broad-ovate, 2–3 × 1–3 cm; margins cartilaginous, veins sparsely setose	**8. *P. cuneifolium***
–	Leaf blades papery; ultimate segments ovate or rhomboidal, 2–7 × 1–3.5 cm; strigose on the veins	**9. *P. davidii***
10	Plant has white lactate	**10. *P. leptophyllum***
–	Plant without white lactate	**11**
11	Stem dwarf; leaves mainly basal, occasionally with 1–2 (–3) heteromorphic cauline leaves; umbels terminal	**12**
–	Stem well developed; leaves basal and cauline, occasionally basal leaves absent; umbels terminal and lateral, occasionally terminal	**17**
12	Leaves heteromorphic; basal ultimate segments flabelliform or lanceolate, cauline ultimate segments lanceolate or elongate-linear	**11. *P. tanakae***
–	Leaves homogeneous, ultimate segments ovate-triangular or linear	**13**
13	Leaves pinnate; ultimate segments ovate-triangular	**14**
–	Leaves ternate 2–4-pinnate or ternate 3–4-pinnate; ultimate segments linear or linear-oblong	**15**
14	Leaves ternate-pinnate; cauline leaf usually absent	**12. *P. subalpinum***
–	Leaves 1-pinnate; cauline leaf 1 or occasionally absent	**16**
15	Leaves ternate 2–4-pinnate; ultimate segments linear	**13. *P. gracillimum***
–	Leaves ternate 3–4-pinnate; ultimate segments linear or linear-oblong, finely dissected	**14. *P. arunachalense***
16	Leaflets (1–) 3–7; umbels 5–8	**15. *P. paucifoliolatum***
–	Leaflets 5–17; umbels 8–20	**16. *P. porphyronotum***
17	Leaves cauline	**17. *P. monophyllum***
–	Leaves basal and cauline	**18**
18	Root tuberous, fusiform	**18. *P. molle***
–	Rhizome distinct	**19**
19	Ultimate leaf segments long-linear, margins entire	**19. *P. caespitosum***
–	Ultimate leaf segments ovate or rhomboidal, margins serrate	**20. *P. botrychioides***

## Taxonomic treatment

### Pternopetalum paucifoliolatum

Taxon classificationPlantaeApialesApiaceae

J.F. Ye, X.Jie Li & Ce H.Li sp. nov.

928DB6A6-D337-5CF3-9521-9C87C9CE2E95

urn:lsid:ipni.org:names:77212875-1

Figs 1 , 2 , 3

#### Type.

China. Sichuan, Emeishan County, Sixigou Scenic Area (29.40°N, 103.38°E), on moist limestone cliff, at an altitude of ca 850 m a.s.l., 02 May 2020, in fruit, *X. J. Li CPG41074* (holotype: PE!).

#### Etymology.

The specific epithet refers to the 3–7 (rare simple) leaflets of this species which differentiate it from all other species of *Pternopetalum*.

**Figure 1.**
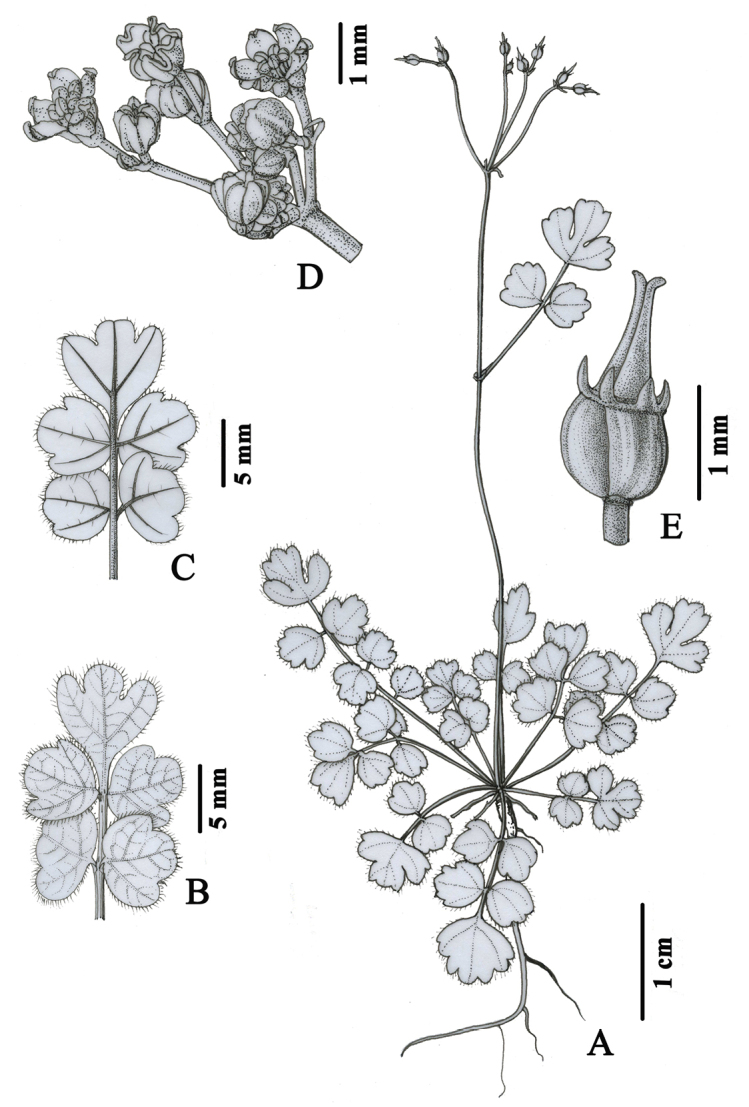
*Pternopetalum paucifoliolatum* J.F. Ye, Xiao-Jie Li & Ce-Hong Li **A** habit **B** adaxial surface of basal leaf **C** abaxial surface of basal leaf **D** umbel **E** mericarp. (Drawn by Y. B. Sun).

#### Diagnosis.

*Pternopetalum paucifoliolatum* differs from *P. porphyronotum* J.B. Tan (2018: e01549) by shorter plants (5–7 cm), 3–7 leaflets (vs. 5–17), leaflet margin white-ciliate, rays 5–8 (Table 1).

Plants 5–7 cm tall. Taproot slender, ca. 3 cm long. Stem 1, unbranched, glabrous. Basal leaves petiolate; petioles 0.7–4 cm; ultimate segments ovate-triangular, 1.5–6 × 0.6–1.4 cm, 1-pinnate, occasionally simple; pinnae 1–3 pairs, broadly ovate, 3–6 × 3–5 mm, lobed, margin white-ciliate, both surfaces glabrous, adaxially green, abaxially purple-red. Cauline leaves 1 or occasionally absent, similar to basal, occasionally linear-lanceolate, 8–12 × 5–6 mm. Umbels terminal, bracts absent, rays 5–8, 10–12 mm, subequal; bracteole 1, linear-lanceolate, 0.5–1 mm; umbellules 2(–3)-flowered; pedicels 0.2–2 mm. Calyx teeth distinct, triangular, ca. 0.3 mm. Petals white, oblong-obovate. Stylopodium conic; style ca. 1 mm, reflexed in the top half, about two to three times as long as the stylopodium. Fruit ovoid, 0.7–1.2 × 0.8–1 mm.

**Table 1.** Comparison of *Pternopetalum paucifoliolatum* sp. nov. and morphologically similar species. Morphological characters obtained from Zhong et al. (2018) and our field observations.

**Character**	***P. paucifoliolatum***	***P. porphyronotum***
Plant height (cm)	5–7	8–15
Stem	1	1, occasionally 2
Basal leaves	1–7, 1-pinnate or simple	5–12, 1-pinnate
Pinnae	ovate-triangular, margin white-ciliate, lobed	ovate-triangular, margins serrate, lacerate-incised or pinnatifid
Adaxial surface	Glabrous	Pubescent
Abaxial surface	purple-red	purple-red (paler on the edge)
Cauline leaf	1, occasionally 0	1, occasionally 0 or 2
Rays	5–8	8–20
Style	about two to three times as long as the stylopodium	approximately twice the length of the stylopodium
Altitude (m)	800–900	1200–1500

#### Phenology.

*Pternopetalum paucifoliolatum* is flowering from March to April, and fruiting from May to June.

#### Distribution and habitat.

*Pternopetalum paucifoliolatum* is only known from its type locality, Sixigou Scenic Area, Emeishan City, Sichuan Province, China. It grows together with grass or mosses on a moist limestone cliff, at 850 m a.s.l. Associated species include *Adiantum* sp., *Begonia wilsonii* Gagnep., *Dryopteris* sp., *Mitreola pedicellata* Benth., *Pteris gallinopes* Ching ex Ching & S. H. Wu., *Pteris* sp., *Selaginella* sp., *Cleistoblechnum eburneum* (Christ) Gasper & Salino, *Viola davidii* Franch.

**Figure 2.**
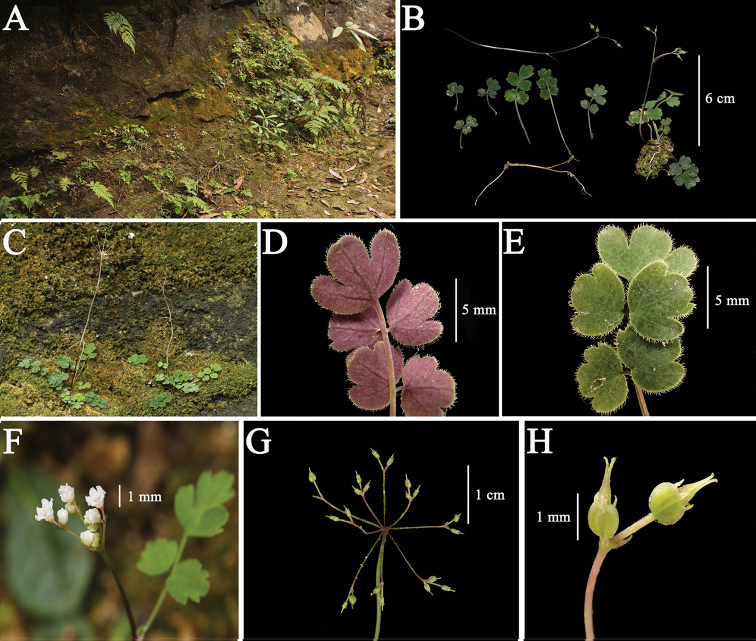
*Pternopetalum paucifoliolatum* J.F. Ye, Xiao-Jie Li & Ce-Hong Li **A** microhabitat **B, C** habit **D** basal leaf blade abaxially **E** basal leaf blade adaxially **F** umbel **G** infructescence **H** umbellule and fruits.

#### Vernacular name.

少羽囊瓣芹 [shǎo yǔ náng bàn qín].

#### Conservation status.

*Pternopetalum paucifoliolatum* is only distributed in Sixigou Scenic Area. We have gone all out to estimate the population size in this area, but found it only growing on one limestone cliff ca 850 m a.s.l., with no more than 200 individuals totally. Sadly, a new built highway will pass by the locality and serves as an entrance to the highway, which will destroy the habitat. Based on this current information and according to IUCN red list criteria (IUCN 2017), *P. paucifoliolatum* should be ranked as ‘Critically Endangered’ (CR C2a (ii)).

#### Discussion.

*Pternopetalum paucifoliolatum* differs markedly from the other known species of this genus by the following characters: basal leaves 1-pinnate, occasionally simple; pinnae 1–3 pairs. It is somewhat similar to *P. porphyronotum* J.B. Tan by 1-pinnate leaves and the abaxially purple-red leaflets, solitary stem and terminal umbel, but differs from it by being 5–7 cm tall (vs. 8–15 cm), stem 1 (vs. 1, occasionally 2), abaxial surface of basal leaves purple-red (vs. purple-red, paler along the edge), rays 5–8, subequal (vs. 8–20, unequal) (Table 1).

**Figure 3.**
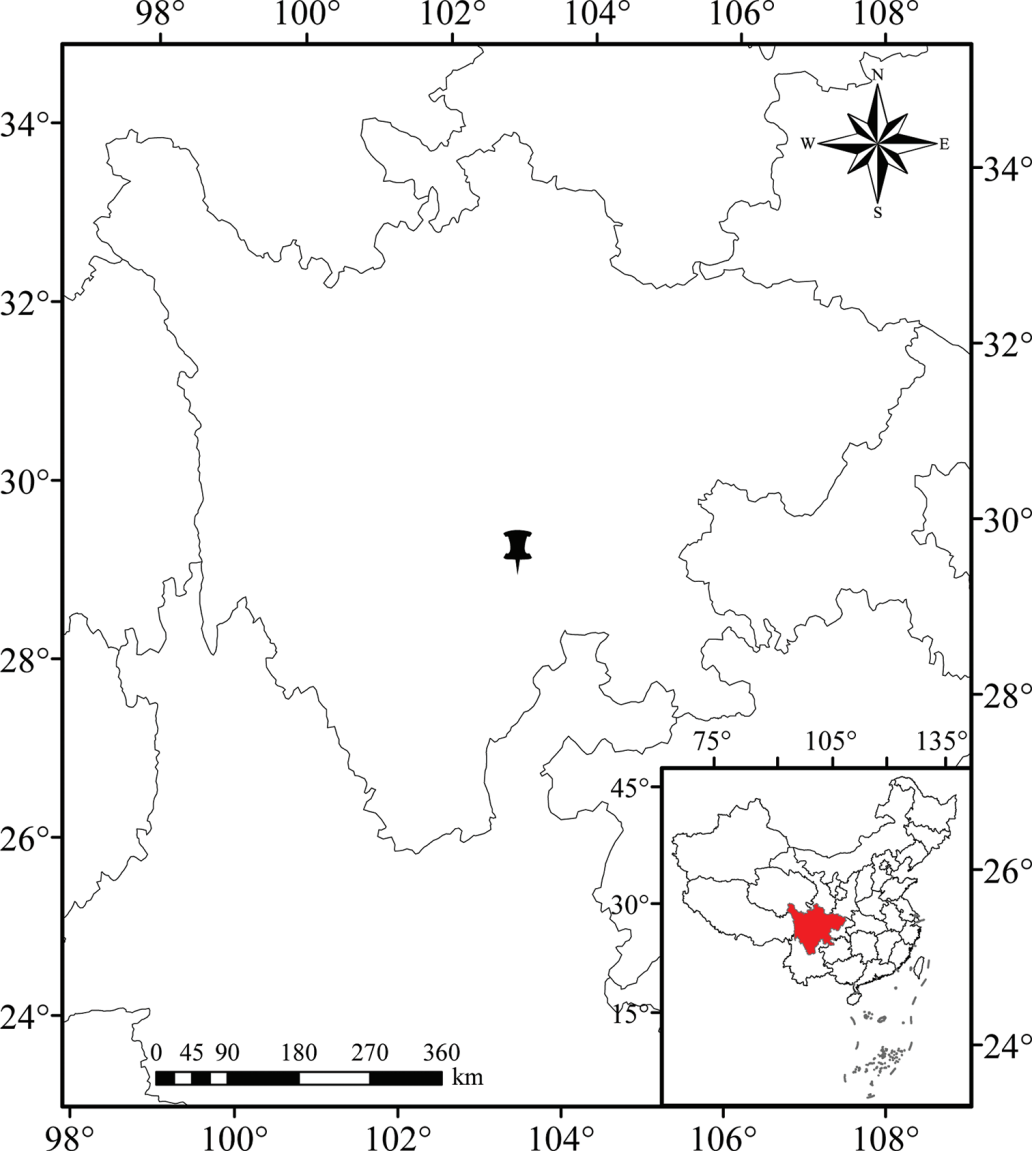
Distribution of *Pternopetalum paucifoliolatum* J.F. Ye, Xiao-Jie Li & Ce-Hong Li in Sichuan Province, China.

## Supplementary Material

XML Treatment for Pternopetalum paucifoliolatum
